# Sustaining self-restraint until the middle of the COVID-19 pandemic in Tokyo

**DOI:** 10.1038/s41598-022-25489-4

**Published:** 2022-12-02

**Authors:** Yoshinao Ishii, Keiichiro Hayakawa, Makoto Chikaraishi

**Affiliations:** 1 Toyota Central R&D Labs., Inc., Koraku Mori Building 10F, 1-4-14 Koraku, Bunkyo-ku, Tokyo, 112-0004 Japan; 2 Hiroshima University, 1-5-1 Kagamiyama, Higashi-Hiroshima, Hiroshima 739-8529 Japan

**Keywords:** Civil engineering, Statistics

## Abstract

We analyzed time-series changes in people’s purpose-specific mobility characteristics owing to the COVID-19 pandemic in the Tokyo area of Japan, where only legally non-binding requests for self-restraint were enforced. A multiple regression analysis was conducted with the objective variable being the mobile population in the Tokyo area per 500 m square grid estimated from mobile spatial statistical data for 2 years from 10/01/2019 to 9/30/2021. This study period ranges from pre- to mid-pandemic. The explanatory variable was the number of buildings by type per 500 m square grid obtained from building statistical data to determine behavioral changes by mobility purpose. The analysis revealed that self-restraint was sustained until the middle of the COVID-19 pandemic in the Tokyo area regardless of the purpose of mobility and whether a state of emergency was declared.

## Introduction

**Figure 1:**
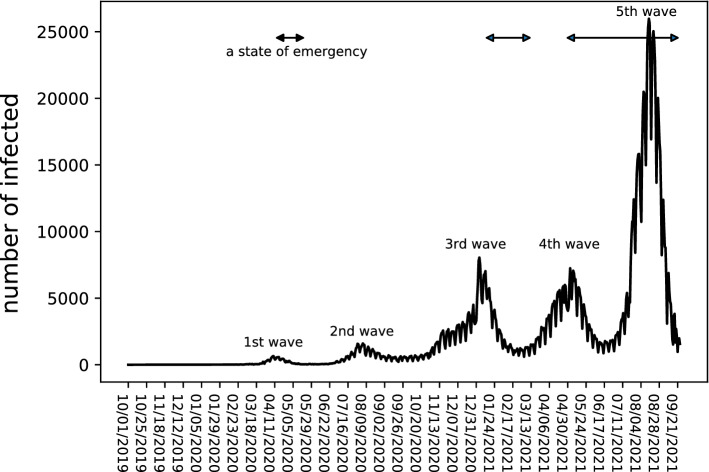
The number of people infected with COVID-19 in Japan. The state of emergencies were declared for the periods indicated by the arrows.

To control the spread of COVID-19, many countries have implemented travel restraint policies with additional reinforcement through strict lockdowns. Legally binding lockdowns have contributed significantly to controlling the increase in the number of infected persons^1–5^. In contrast, Japan follows institutional constraints that do not grant the central government the power to enforce a lockdown^6^. In other words, lockdowns cannot be imposed in Japan. Therefore, in Japan, voluntary travel restrictions were requested by declaring a state of emergency, mainly in urban areas where the number of infected people tends to be higher than in rural areas. Figure 1 depicts the number of COVID-19 cases in Japan and the period during which a state of emergency was declared as the period analyzed by this study^7^. Despite the request for voluntary travel restraint without legally binding force, existing studies have shown that the mobility behaviors of people in Japan have changed significantly compared to the pre-COVID-19 period^8–20^. It is important to understand how the voluntary lockdown has changed travel behaviors to determine urban development and transportation policies during and after COVID-19.

Several studies in recent years have examined mobility changes in Japan using location data collected from mobile phones^9,10,14,18–22^. Studies of mobility change based on web-based questionnaires can reveal detailed changes in mobility characteristics over a specific period^8,15,23^ while studies using mobile phone data can reveal sequential mobility changes. Although there are multiple sources of mobile data used to study mobility change^9,18,21,24,25^, mobile spatial statistics (MSS) data^26^ are commonly used in Japan^10,14,19,20,22^. MSS data are population statistics created from the operational data of mobile terminal networks provided by NTT docomo, which is the largest carrier in Japan, holding 37.5% of mobile phone contracts^10^. The mobile population within each spatial grid (typically a 500 m square grid) estimated using MSS data has high spatio-temporal coverage and high update frequency, making it suitable for analyzing mobile change during the COVID-19 pandemic in Japan. Previous studies have revealed statistical mobility changes in people before and after the COVID-19 pandemic. For example, an increase in the number of infected people significantly reduced long-distance travel and travel to congested places^20^, and the first wave of the pandemic receded because of a significant decrease in travel as a direct result of the request for self-restraint^19^. Studies using Japan’s MSS data have provided detailed analyses of mobility changes that cannot be revealed by aggregated data such as the Google Mobility Report^21^, which provides an excellent overview of behavioral change. However, these studies have not revealed changes in mobility by purpose of travel because the MSS data contain no information other than that related to population.

In this study, we reveal mobility change by mobility purpose in the Tokyo area through a combined analysis of MSS data with a 500 m square grid resolution for 2 years from 10/01/2019 to 9/30/2021. This period includes the pre-pandemic period and the fifth wave of infection explosion. Our analysis also includes building statistics data^27^, which hold the number of buildings by type per 500 m square grid, provided by Zenrin Marketing Solutions. In the field of traffic engineering, land use and building types are generally accepted to generate mobility with a corresponding travel purpose^28–30^. Therefore, the number of buildings per cluster, which is obtained by clustering buildings, rooms, and offices by type in a specific grid, is a feature that generates purpose-specific mobility in that grid. We apply a multiple regression analysis using the total weekly mobile population in each grid as the objective variable and the number of buildings by type (e.g., commercial, school, and restaurant) in each grid as the explanatory variables, in chronological order, to analyze changes in the coefficients over time. This allows us to reveal how mobility by purpose of travel (e.g., commuting to work, going to school, eating out) has changed with the expansion of the COVID-19 pandemic. In particular, the smaller value of a coefficient after compared to before the COVID-19 pandemic indicates that mobility for the corresponding travel purpose has been restrained.

Through the above analysis, we identify time-series changes in purpose-specific mobility after the COVID-19 pandemic in the Tokyo area. In particular, this study aims to determine whether travel self-restraint was sustained for each purpose of travel. Detailed analysis of the sustained self-restraint from this study will contribute to future urban development and transportation policy decisions in Japan’s urban areas.

## Results

**Figure 2:**
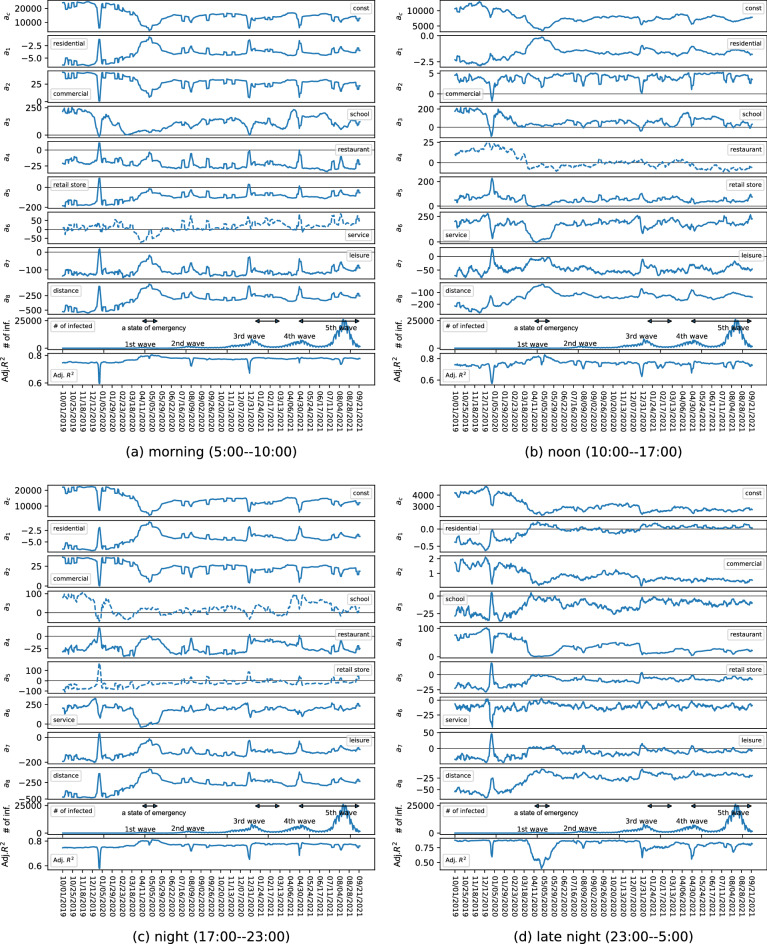
Time variation of coefficients for each time period over 2 years.

Figure 2 shows not only the time variation of the partial regression coefficients and the adjusted R-squares (Adj. R$^2$) for each period but also the number of COVID-19 cases throughout Japan. Figure 2a–d show the results for the morning, afternoon, night, and late-night hours, respectively. The vertical axes indicate the values of each coefficient/variable, and the horizontal axes indicate the date. In particular, the vertical axes for $a_1, \ldots , a_8$ represent the impact on the mobile population per unit number/distance and that for $a_c$ represents the constant term. $a_1,\ldots ,a_8$ represent the coefficients for residential ($a_1$), commercial ($a_2$), school ($a_3$), restaurant ($a_4$), retail store ($a_5$), service ($a_6$), leisure ($a_7$), and distance to the central business district ($a_8$), respectively. Note that each coefficient corresponds to the impact of each travel purpose on the travel volume. For example, commercial corresponds to commuting, school corresponds to going to school, restaurant corresponds to eating out, and so on. A smaller value of a coefficient after compared to before the COVID-19 pandemic suggests that mobility for the corresponding travel purpose was restrained. The coefficients with solid lines indicate that the median P value was less than 0.05, suggesting that the result was statistically significant. In contrast, the coefficients written in dotted lines are those for which the median P value in all windows was greater than or equal to 0.05, indicating that the results were statistically insignificant. For coefficients that changed positively or negatively during the period, a horizontal line is drawn where the value is zero, and the figure for the number of infected people shows the period when a state of emergency was declared^7^.

The results are analyzed below. First, the adjusted R$^2$ values were found to be high for all periods, confirming that the extent of human mobility is well represented by the number of buildings by type. The large drop in adjusted R$^2$ around New Year’s Day in 2020 may be because of the temporary closure of stores and people returning to their home towns.

Second, we investigated the mobility change in the vicinity of the first state of emergency declaration (April 7–May 25, 2020)—that is, during the first wave of the COVID-19 pandemic, when the absolute values of the partial regression coefficients for most of the features were smaller than those before the pandemic in all periods. This may indicate that the impact of each feature on the mobile population decreased because of the sense of crisis caused by the pandemic or the self-restraint with regard to telecommuting and long-distance travel owing to the declaration of a state of emergency. This result is consistent with the results of a study based on a web-based questionnaire survey^15^. One notable change during this period is that of “restaurant” in the late-night hours. Before the pandemic, “restaurant” was the feature that strongly influenced the increase in late-night travel, and this was considered to be dominated by drinking establishments. However, the influence of “restaurant” on the increase in travel was significantly reduced due to the self-restraint of night-time business operations following the declaration of the state of emergency. The effect of restaurants in the late-night hours on the mobile population declined with each emergency declaration, even after this period, confirming that the request for shorter business hours due to the state of emergency declarations affected the reduction of the mobile population in the late-night hours. The change in the coefficient of “school” during the morning and afternoon hours began to decrease from a very early stage, unlike the other coefficients. This may reflect the fact that many schools were temporarily closed at an early stage when COVID-19 cases began to be confirmed.

Third, we examined the mobility change from after the first wave to before the third wave (around December 2020). After the first wave subsided and the state of emergency was lifted, the absolute values of the partial regression coefficients for all periods increased, but the magnitude remained smaller than before the pandemic regardless of the number of infected people. This may be attributable to the fact that people became accustomed to refraining from long-distance travel because of the spread of telecommuting and continued practicing voluntary restraint in personal travel. It can also be confirmed that, in the second wave (around July–September 2020), the change in mobility remained smaller than in the first wave. This result is consistent with the findings of existing studies^19,20^. Note that the variation of the partial regression coefficient and the adjusted R$^2$ in August may be largely attributable to the summer vacation.

Fourth, we analyzed the mobility change from the third wave to the period after the convergence of the fourth wave (December 2020–June 2021). During this period, the magnitude of the partial regression coefficients for each feature remained smaller than before the pandemic regardless of the number of infected people and whether a state of emergency was declared. For both the third and fourth waves, the absolute values of the partial regression coefficients decreased significantly when the number of infected people reached its peak. In contrast to the first wave, we can confirm that these values immediately return to the same values as before the second wave and remained unchanged regardless of the declaration of a state of emergency. Therefore, while mobility was temporarily greatly suppressed during the peak period, once the number of infected people began to decrease, the population continued to follow a certain degree of voluntary restraint in their mobility regardless of the issuance of state of emergency declarations. This may be because people became accustomed to the state of emergency declarations and the COVID-19 pandemic and because of self-restraint fatigue. The results of this analysis are consistent with a study of inter-prefectural travel change using extensive mobility data from the same period in Japan as in this study^18^.

Fifth, we examined the mobility change around the fifth wave (after July 2021). Here, the number of infected people was larger than before, but the coefficient change was generally smaller than in the first, third, and fourth waves. Therefore, it can be confirmed that general mobility restrictions during the COVID-19 pandemic were in place during the fifth wave, although the restrictions imposed were not particularly strong. Nevertheless, the decrease in the number of infected people can be attributed to factors such as increased vaccination rates, although further investigation is needed to determine the cause.

Finally, we analyzed the overall changes before and after the COVID-19 pandemic. In this study, the analysis was conducted up to the fifth wave of the COVID-19 pandemic, and the absolute values of the coefficients remained smaller than those before the pandemic for almost all periods and travel purposes. This decrease in the absolute value of the coefficients appears to have been sustained for most of the features regardless of whether a state of emergency was declared. This indicates that the decline in human mobility was well-established in the urban areas of Japan even without legally binding lockdowns. Therefore, it can be concluded that self-restraint was sustained until the middle of the COVID-19 pandemic in urban Japan regardless of the mobility purpose and whether a state of emergency was declared.

## Discussion

We analyzed changes in people’s behaviors due to the COVID-19 pandemic by purpose of travel using multiple regression analysis. The objective variable was the mobile population calculated based on 2 years of MSS data for urban areas before and after the pandemic. The explanatory variables were features such as building type and distance to the central business district. The results of the multiple regression analysis revealed that the mobile population decreased significantly in the first wave of the COVID-19 pandemic over a prolonged period, regardless of the purpose of travel. In the second and subsequent waves, although the mobility restraints against the infectious explosion became more modest than in the first wave, irrespective of the purpose of travel, we confirmed that voluntary mobility restraints became entrenched, regardless of the number of infected people. This suggests that self-restraint was sustained until the middle of the COVID-19 pandemic in urban Japan. This sustained self-restraint may be attributable to changes in the social system, such as the pervasiveness of telecommuting and the diffusion of delivery services or the internalization of new social norms.

Our analysis of the overall reduction in people mobility due to the COVID-19 pandemic is consistent with a number of existing studies, as is our analysis of the mobility changes due to multiple state of emergency declarations. What is new to our study is our finding, with statistical support, that self-restraint in mobility was maintained until the middle of the COVID-19 pandemic irrespective of the purpose of mobility. This finding is a new contribution to the formulation of future urban development and transportation policies.

This study is subject to certain limitations. First, the MSS data used in this study lacked sufficient spatio-temporal coverage owing to our monetary restrictions. Therefore, we were unable to determine whether self-restraint was sustained in Japan as a whole and whether it continued after the period for which the data were used in this study. Second, several potential coverage biases have been noted in the cell phone data^31^. In this study, age bias in particular may have influenced the results^32^; however, the unavailability of age information precludes the discussion of differences in mobility change across generations. Third, the building statistical data only covered 2020 owing to our monetary restrictions, which may have introduced noise in the coefficients for 2021^33^.

Despite the above limitations, our results provide significant implications for formulating urban policies. In Marshallian economics, agglomeration effects have been divided into technological spillovers, labor pooling, and intermediate input linkages while such effects would change over time depending on the development of information and communication technologies^34^. In general, such technologies may gradually change urban structure while our results suggest that the COVID-19 has accelerated such a structural change. If this is the case, it may be necessary to revisit a number of urban policies that rely on the conventional agglomeration forces such as concentrating offices in a particular area. It will be important to continue to examine whether the self-restraint will be sustained in the future, and urban policies should be adapted to the actual conditions of mobility.

## Methods

### Mobile population data

In this study, MSS data for the Tokyo area were used to generate mobile population data. The MSS data comprise the data of the estimated population staying within a certain area at a certain time based on the mobile phone base station access data provided by NTT Docomo, a Japanese mobile phone operator. The resolution of the MSS data used in this study reflects a 500 m square grid (also called a quadratic grid) that has an hourly estimated population. We used the MSS data with this resolution for 940 grids over 2 years, from 10/01/2019 to 9/30/2021. The locations of the 940 grids were selected as follows to provide diversity:

- With the Imperial Palace as the urban center, we calculated the distances between the center of gravity of each municipality and the Imperial Palace.
- To analyze the relationship between the distance from the urban center and the mobile population, municipalities within five ranges of distance from the Imperial Palace (0–5 km, 5–15 km, 15–25 km, 25–35 km, and 35–45 km) were selected.
- From the municipalities within each range, four were selected, two each with the highest and lowest population densities, to ensure diverse data.
- To avoid selecting grids with little or no human mobility, from each of the selected municipalities, the grids with the top 50 highest population densities were selected.

**Figure 3:**
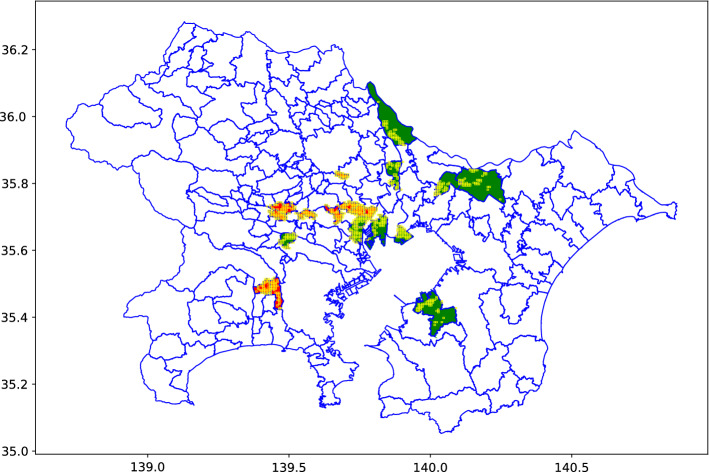
The distribution of the grids used in this study. The vertical axis, horizontal axis, and yellow squares represent the latitude, longitude, and grids used in this study, respectively. The red and green represent the municipalities with high and low population densities, respectively.

For procedure (c), mixing areas with high and low population densities can generally degrade the quality of the analysis when performing a spatial change analysis whereas, for the temporal change analysis in this study, the mixed areas chosen for this procedure cover a wide variety of spaces. The municipalities thus selected are depicted in Table 1, and the distribution of grids is depicted in Fig. 3. The number of grids is 940 instead of 1000, or 20 (the number of municipalities) $\times$ 50 (the number of grids), because Warabi-city has only 34 grids in the whole area, and the same grids are adopted in adjacent municipalities. The full urban area data could not be used owing to our monetary limitations.

**Table 1:** Municipalities with the two highest/lowest population densities in each range.

range	Top 1	Top 2	Bottom 1	Bottom 2
0–5 km	Bunkyo-ku	Taito-ku	Chiyoda-ku	Minato-ku
5–15 km	Toshima-ku	Nakano-ku	Urayasu-city	Ota-ku
15–25 km	Warabi-city	Musashino-city	Misato-city	Inagi-city
25–35 km	Kokubunji-city	Kodaira-city	Noda-city	Shirai-city
35–45 km	Yamato-city	Zama-city	Sodegaura-city	Inzai-city

Based on the above 940 grid-by-grid and hourly population estimate data, the hourly mobile population for each grid is estimated. The following method is used to estimate the mobile population from the population data based on the method proposed by Hara et al.^14^. If $p_{g,t}$ is the estimated population at time *t* of a grid *g*, the estimated mobile population $y_{g,t}$ at *t* of *g* is calculated as follows:

1 $$\begin{aligned} y_{g,t} = |p_{g,t} - p_{g,t+1}| \end{aligned}$$

Although the estimated mobility population obtained by Eq. (1) is lower than the actual mobility population, this estimation method can be used for the analysis of mobility trend changes, as shown by Hara et al.^14^ and Tsuboi et al.^20^.

### Data on number of buildings by type

This section describes the data used as explanatory variables in the regression analysis. The number of buildings, rooms, and offices in the grid and the distance of the grid to the Imperial Palace were used as explanatory variables. In the field of traffic engineering, land use and building types are generally accepted to generate travel with a corresponding travel purpose^28–30^. For example, a school generates travel for the purpose of commuting to and from school, and a restaurant generates travel for the purpose of eating and drinking. Therefore, the number of buildings per cluster, which is obtained by clustering buildings, rooms, and offices by type in a specific grid, is a feature that generates purpose-specific travel in that grid. The number of buildings belonging to a given cluster (e.g., residential or commercial) was considered an explanatory variable for each grid. The distance to the Imperial Palace was also employed as an explanatory variable to analyze the effect of the difference between urban and suburban areas. We used the 2020 version of the building statistics data^27^ provided by Zenrin Marketing Solutions to count the buildings by type. The building statistics data include information such as the number of detached houses and the number of restaurants within a 500 m grid, similar to the MSS data. In this study, the eight types of features shown in Table 2 were used as explanatory variables. Each feature was the sum of the number of buildings by cluster shown in the second column of Table 2 and was denoted by $x_1, \ldots ,x_8$.

**Table 2:** Features to be used as explanatory variables.

Feature (notation)	Types (total number)
Residential ($x_1$)	Detached houses, houses with offices and residences, rooms in apartment houses, rooms in multi-family buildings, rooms in dwellings
Commercial ($x_2$)	Offices
School ($x_3$)	Buildings related to education
Restaurant ($x_4$)	Restaurants
Retail store ($x_5$)	Food stores, clothing stores, daily goods stores
Service ($x_6$)	Stores related to rentals, weddings, funerals, lifestyle, automobiles, others
Leisure ($x_7$)	Sports facilities, entertainment facilities, hotels, inns
Distance ($x_8$)	Distance between urban center and grid center

### Analysis method

**Figure 4:**
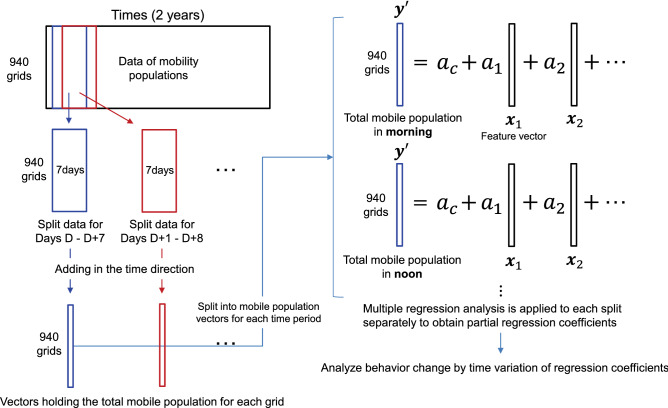
An overview of the analysis method.

Using the mobility population per grid as the objective variable and the features as the explanatory variables, the coefficients for each explanatory variable were calculated and analyzed using multiple regression analysis. The variance inflation factors for the eight variables were 2.09, 6.18, 1.54, 2.90, 4.51, 3.07, 2.56, and 2.29. Therefore, the magnitude of multicollinearity was acceptable. Figure 4 presents an overview of the analysis method. To examine the variation of the coefficients within the period, we split the data with a window width of 7 days and a sliding width of 1 day. This produced 725 pieces of split data. Furthermore, as people’s mobility is considered to differ greatly depending on the time of day, we divided each of the 725 pieces of split data into four periods: morning (5:00–10:00), afternoon (10:00–17:00), night (17:00–23:00), and late night (23:00–5:00). Multiple regression analysis was applied separately to these 725 $\times$ 4 weekly and hourly split data to analyze the time variations of the coefficients per period. The following multiple regression model was used:

2 $$\begin{aligned} {\varvec{y}'} = a_{c} + a_{1} \varvec{x}_{1} + \cdots + a_{8} \varvec{x}_{8}, \end{aligned}$$

where ${\varvec{y}'}$ is a 940-dimensional vector representing the total mobility population number per grid by adding the split mobility data in the time direction, $\varvec{x}_i$ is a 940-dimensional vector holding the feature value $x_i$ for each grid, $a_{c}$ is a constant term, and $a_i$ is the partial regression coefficient on $\varvec{x}_i$. The coefficients for the split data were determined to minimize the squared error on both sides of this model (2).

## Data Availability

The data that support the findings of this study are available from NTT Docomo and Zenrin Marketing Solutions but restrictions apply to the availability of these data, which were used under license for the current study, and so are not publicly available. Data are however available from the authors upon reasonable request and with permission of NTT Docomo and Zenrin Marketing Solutions.
